# The key issues and development strategy of Chinese Classical Formulas pharmaceutical preparations

**DOI:** 10.1186/s13020-021-00483-6

**Published:** 2021-08-04

**Authors:** Hua Luo, Hongguo Chen, Chang Liu, Siyuan Zhang, Chi Teng Vong, Dechao Tan, Yuntao Dai, Yitao Wang, Shilin Chen

**Affiliations:** 1 Macau Centre for Research and Development in Chinese Medicine, Institute of Chinese Medical Sciences, University of Macau, Macao, China; 2 Key Laboratory of Beijing for Identification and Safety Evaluation of Chinese Medicine, Institute of Chinese Materia Medica, China Academy of Chinese Medical Sciences, Beijing, China

**Keywords:** Chinese Classical Formulas, Material Reference, Origin of the medicinal materials, Processing methods, Dosage verification, Quality control, Development strategies, Safety evaluation

## Abstract

It is well-known that Prof. Tu Youyou won the Nobel Prize in Physiology or Medicine in 2015 due to the research on artemisinin treating malaria, and this can be regarded as the milestone of modernization of Traditional medicine. This first Nobel Prize in Traditional Chinese medicine (TCM) has aroused profound impetus in the investigation of TCM and attracted global attention to the ancient books of TCM. Three new medicines for the treatment of COVID-19 derived from Chinese Classical Formula (经典名方, CCF) have been approved in 2021 due to their effectiveness for the treatment of COVID-19. This article introduced the research background of CCF pharmaceutical preparation (CCFPP), explained the ideas for the modernization of CCF and analyzed related issues involved in the development process of CCFPP, including the origin of medicinal materials, processing methods, dosages and the preparation process of CCF Material Reference. The strategy for industrialization was proposed in terms of the evaluation of the pharmaceutical properties, industrialization considerations, and clinical positioning of CCFPP. The key contents and requirements for the development CCFPP were also summarized according to the recently published registration guidance by the Center for Drug Evaluation in China. In addition, the safety issues of CCFPP were described, including the discussion on the non-clinical safety evaluation and analyzation on the international registration of Traditional herbal medicines. This article is aimed to provide references for enterprises, researchers, and relevant personnel of government departments that are engaged in the development of CCF to speed up the developing process of CCFPP.

## Introduction

Traditional Chinese medicine (TCM) has made outstanding contributions to our health and the treatment of many diseases, and this causes a global developmental upsurge. The research by Prof. Youyou Tu on the research of artemisinin treating malaria gained a Nobel Prize in Physiology or Medicine in 2015. As the milestone of the modernization of Traditional medicine, it has attracted global interests to many scientists on the investigation of TCM [1]. As the coronavirus disease 2019 (COVID-19) pandemic causes a significant global public health problem, TCM was used as a treatment for 91.5% of the COVID-19 cases in China, which showed promising results in improving symptoms and reducing disease deterioration, mortality and recurrence rates [2]. Three formulas (Qingfeipaidu Decoction, Huashibaidu Decoction and Xuanfeibaidu Decoction) developed from Chinese Classical Formula (经典名方, CCF) have been selected as general effective medicines for the treatment of COVID-19 and approved as new medicines in 2021 [3].

CCF are the prescriptions recorded in the medical records of the Qing Dynasty and before the Qing Dynasty and are still widely used, and they have definite curative effects and obvious characteristics and advantages [4]. The list of CCF should be promulgated by Chinese government and 100 CCF was promulgated in 2018 [5]. CCF are essential and supportive for the development of prescription theory. The prescription theory is based on the system of "principles, methods, formulas and medicine" (理法方药). After nearly two thousand years of continuous practical use, analysis, improvement and complement on theories, a tremendous amount of formula/prescription-related records have been accumulated. Some formulas with more precise therapeutic effects are widely used at a higher priority and considered as classical formula/prescription for the treatment of specific diseases. CCF are the essential formulas that have accumulated practical experience in the clinical application of Chinese medicine since thousands of years ago. With their remarkable therapeutic effects, they are currently still in use by medical doctors. It is a vital strategy to transform these Chinese treasures represented by CCF into high-quality products for easy to carry and use, which is a preferred shortcut for developing TCM products and meets the needs of diversified medications.

The development of CCF pharmaceutical preparations (CCFPP) is a "green channel" for the drug development in China. Due to the complexity of TCM and its weakness in basic research for modernization, the success rate of new drug development from TCM is relatively low. Only five new drug applications were approved during 2014 to 2019, while forty new drug applications were approved from 2007 to 2013 [6]. The document "Opinions of the State Council on Reforming the Review and Approval System for Drugs and Medical Devices" [7] followed a series of reform policy documents that led to a sharp decrease in the number of new drug applications. The development of CCFPP has provided good ideas and opportunities for the development of new drugs from TCM. The core idea of CCF development is to refine the application of ancient and modern clinical practice and use it to design modernized pharmaceutical preparations with stable quality. For the application of CCFPP registration, the core advantage is that pharmacological research and clinical trial data are not needed. The research period of CCF is considerably shortened and the cost is drastically reduced.

The development and research of CCFPP have attracted a lot of attention to scientists, which has caused a large number of enterprises and researchers to invest in it. Meanwhile, the preliminary research, production and regulatory practice of China's formula granules (配方颗粒) have also provided a lot of experiences and references for the development of CCF in the past two decades.

The new version of the “Drug Administration Law” implemented in 2019 in China requires systematic research, quality control based on the whole manufacturing chain, quality traceability and adverse reaction monitoring for drug registration, which put forward new requirements for drug registration. The new law also emphasizes the responsibility of enterprises and other entities. There are still many gaps where policies have not yet been implemented for some specific CCF varieties. Although the authoritative role of expert consensus has been clarified, the specific determination criteria are still incomplete. The risks of CCF development are still high with policy factors, market capacity and uncertainty in the research process. Therefore, this study, was aimed to provide references for enterprises, researchers and relevant personnel of the governmental departments that are engaged in the development of CCF to speed up the developing process of CCFPP.

## Ideas for the development of CCFPP

*Simplified Regulations on the Administration of Registration and Approval of Traditional Chinese Classical Formula Pharmaceutical Preparations (CCFPP*) (referred to as *Registration Regulations CCFPP*) pointed out seven conditions that should be met for the application of CCFPP, which can be exempted from performing clinical trials [8]. When CCFPP meet the listed requirements, they can provide pharmaceutical and non-clinical safety research data for the registration of CCFPP without pharmacological research and clinical trial data. These seven regulations exclude the highly toxic Chinese medicine materials, thereby ensuring their safety. Besides, these regulations also limit the consistency of the formula composition, preparation method and administration methods recorded in ancient books of TCM, thereby ensuring the effectiveness and safety of the CCFPP.

### Two stages for the development of CCFPP

**Fig. 1:**
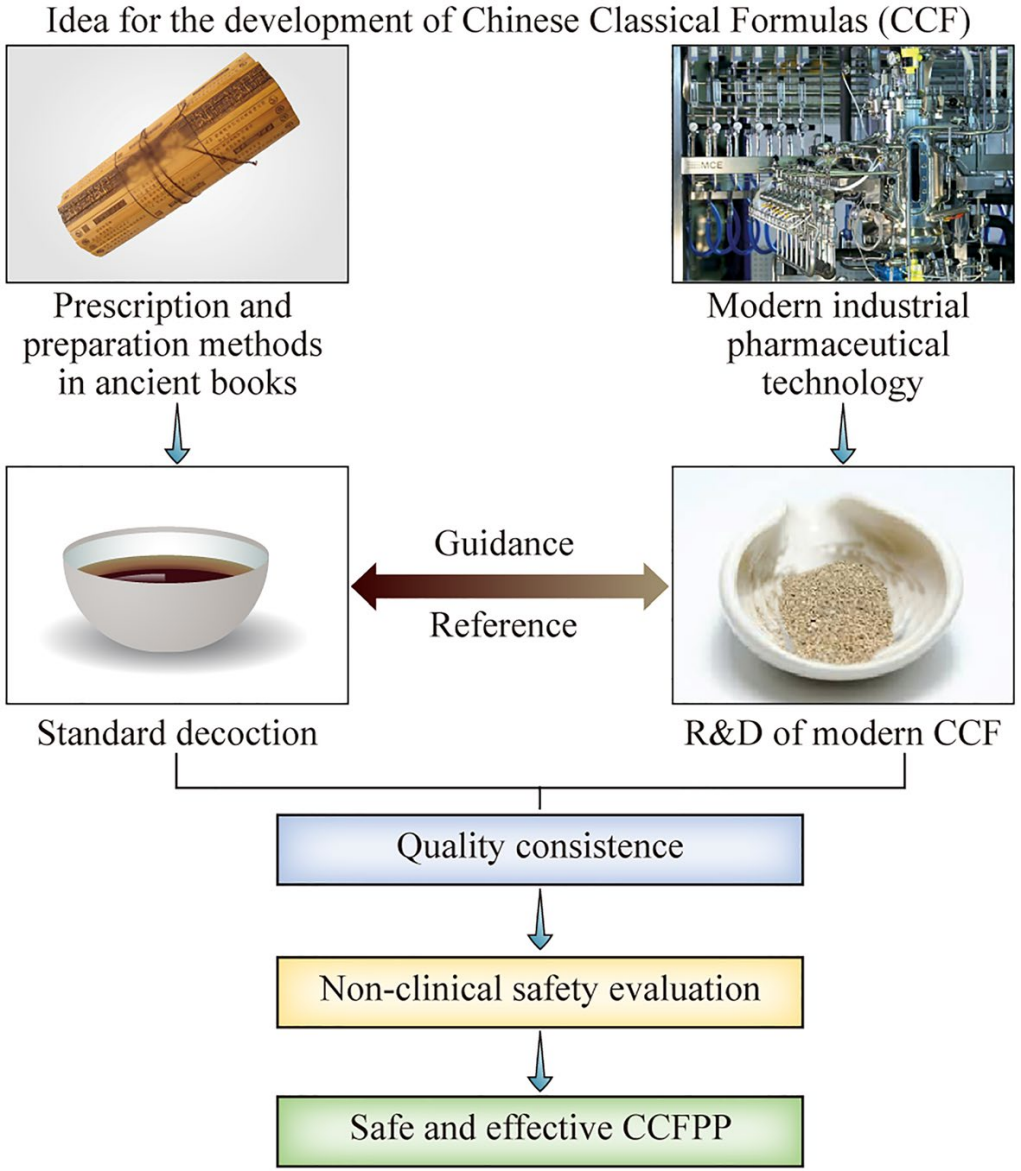
The guidance for the development of Chinese Classical Formula pharmaceutical preparations (CCFPP). The standard decoction or the material reference of CCF is a reference for the optimization of the production process and quality evaluation of the CCFPP

The *Registration Regulations of CCFPP* pointed out that the development of CCFPP is divided into two stages: the research and development (R&D) of the CCF Material Reference (MR, 物质基准) and the R&D of pharmaceutical preparation based on the CCFMR to ensure that the key quality attributes of CCFMR and CCFPP are consistent [8] (Fig. 1). The CCFMR is a reference for the production process optimization and quality evaluation of the CCFPP. It refers to the standards of medicinal substances in CCF that are prepared based on the preparation methods recorded in the ancient medical books, and it is called standard decoction (标准汤剂) for water decoction preparation. It is an intermediate reference for the transformation of TCM from traditional individualized preparation to industrial pharmaceutical products. The involvement of CCFMR in the development of CCF ensures that the clinical efficacy of the pharmaceutical preparations is not decreased, their toxicity is not increased, and their product quality is stable and consistent.

To further ensure the safety of CCFPP, non-clinical safety studies with Good Laboratory Practice (GLP) specifications should be done for the application of the CCFPP according to the *Registration Classification and Application Requirements for Traditional Chinese Medicines* [9] and the *Registration Regulations of CCFPP*. Non-clinical safety research together with sufficient clinical practical experience of CCF can further ensure the safety of CCFPP.

### The development of CCFMR

The concept of CCFMR has gone through a long period of demonstration. The concept of standard decoction (标准汤剂) was first proposed for the development of Japanese Kampo (汉方制剂) in 1986 [10]. The standard decoction is the chemical and biological base for the development of Japanese Kampo to ensure good product quality. Because Japanese Kampo preparations are mainly derived from CCF, the concept of "standard decoction" proposed by Japan is worth learning for the industrialization of CCF in China. Therefore, the concept of "standard decoction" was introduced as a reference for production and quality management of CCFPP. In April 2016, Prof. Shilin Chen proposed the concept of "standard decoction of medicinal material decoction pieces" [11, 12]. In August 2016, the concept of "standard decoction" was applied in the quality control of granules of decoction pieces in the *Technology for Quality Control and Standard-setting of Traditional Chinese Medicine Granules of Pieces (Draft for Solicitation of Comment*s) promulgated by the National Pharmacopoeia Commission [13]. Subsequently, the National Medical Products Administration in China (NMPA) adopted the concept of standard decoction in the *Registration Regulations of CCFPP (Draft for Comment*s*)* promulgated in October 2017. The concept of CCFMR was then adopted in the *Registration Regulations of CCFPP,* which was finally promulgated in June 2018. Although the name of CCFMR has been changed to different names, such as "standard decoction", "standard Jianye", "key samples", but their essence is the same, namely the reference for CCFPP.

## Key issues for the establishment of CCFMR

**Fig. 2:**
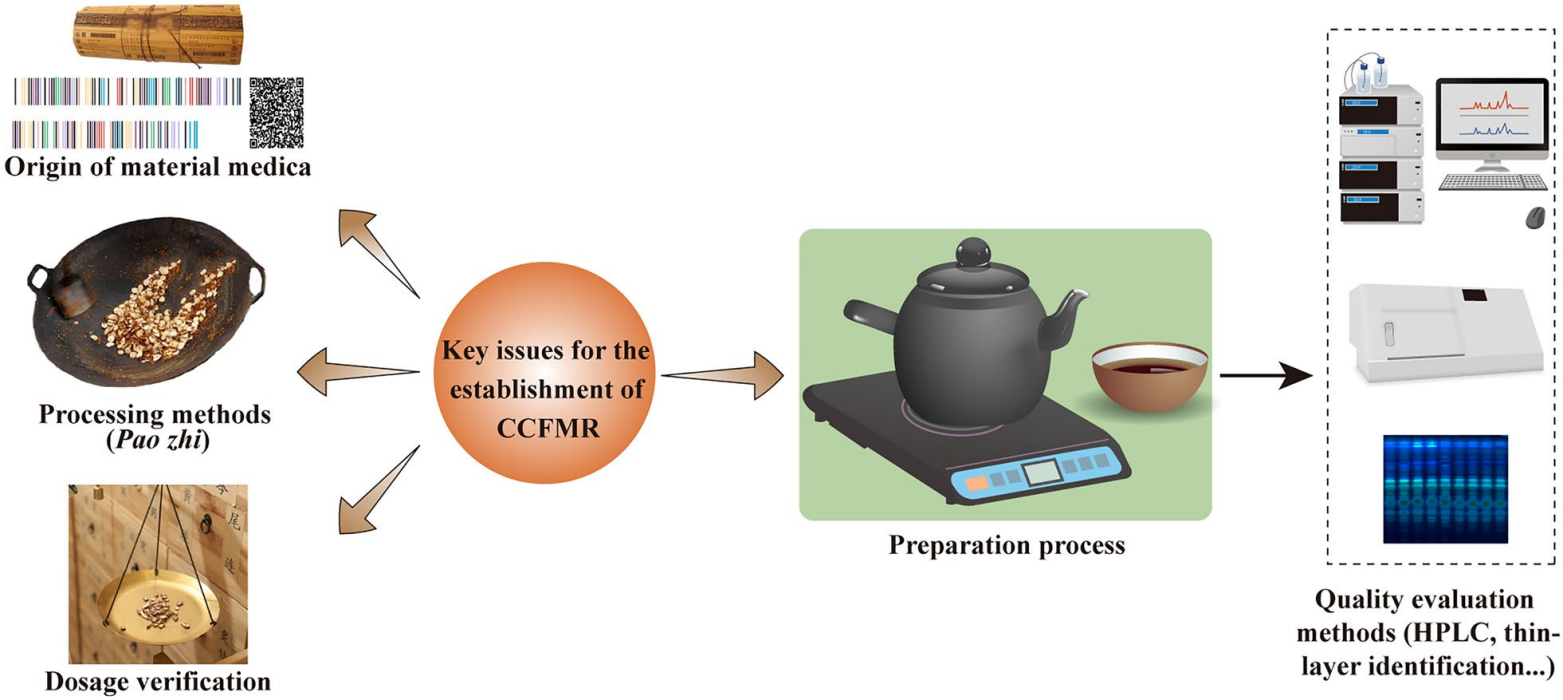
Key issues for the establishment of Material Reference of Chinese Classical formula (CCFMR). The preparation of MR is based on the preparation methods recorded in the ancient books, but there are four main issues, including the origins of the medicinal materials, processing methods of the decoction pieces, dosages, and the preparation methods

The preparation of CCFMR is based on the preparation methods recorded in the ancient medical books but it is a big difficulty in developing CCF. There are no success cases for referencing, and the officials has not published declaration guidelines yet. There are also uncertainties in relation to policies, healthcare market and R&D process. There are four main issues, including the origins of the medicinal materials, processing methods of the decoction pieces, dosages, and the preparation methods of the CCFMR (Fig. 2). These key common issues are needed to be verified by comprehensive consideration of the ancient records with modern clinical applications, and some of them may need be confirmed by the scientific research evidence. The quality of CCFMR is also needed to be evaluated with modern quality control methods, which is important as a reference for the R&D of CCFPP [14].

### Origin of the medicinal materials

“Origin” is the systematic summary of the species, effective parts, and habitat of the medicinal materials. The varieties and medicinal parts of some Chinese medicine have changed with time due to the facts that it has been used for a long history of thousands of years and the original plants of the same family and genus have similar morphology of the variety of species, and some records on the morphology given by the ancient books are too simple. Thus, it is necessary to clarify the historical origins and changes of the source of Chinese medicine material through the research on origin to determine their varieties and medicinal parts for the development of CCF, and to accurately pass the medical experience to others and ensure good safe use of medicine. There are 159 types of Chinese medicine materials involved in 100 CCF. The medicinal parts comply with the Chinese Pharmacopoeia (ChP) for most of the Chinese medicine materials, and most of those are from their original habitat [15]. An essential purpose in the research of origin is to identify species and avoid using substitute and adulterated medicine. Chinese medicine substitutes refer to the medicine with the same taste, meridian entry (归经), function and indications as the original Chinese medicine. Chinese adulterated medicine refers to the Chinese medicinal products whose sources or medicinal parts do not meet the criteria of ChP or other statutory drug standards, including the confusion of species, medicinal parts, and intentional fraud. Chinese medicine substitutes and adulterated medicine can interfere with the correct usage of the original Chinese medicine. To avoid the influences by the substitute and adulterated medicines, researchers will use textual research of the ancient records to trace the change of their origins and combine this with modern technology, like DNA barcoding [16], trait identification [17], or Q-marker to identify the original Chinese medicine materials [18].

Several main methods for the textual research of origin are Materia Medica Research, Botanic (animal, mineral) Taxonomy Research, Molecular Identification Research, and Chemical Identification Research. Materia Medica Research reviews the literature on material medica and its traits to verify the name, origin, and habitat of different Chinese medicine materials in each era. The basic operation process of the textual research on material medica includes the acquisition of drug characteristic information and proof of the origin. The acquisition of characteristic information mainly includes three key phases: reviewing, sorting out and selecting literatures. The molecular identification of Chinese medicine is mainly based on the characteristics of macromolecules (protein and nucleic acid), which can be divided into protein and nucleic acid identification. At present, the main methods of molecular identification of Chinese medicine are DNA barcode identification, specific polymerase chain reaction method, population genetic analysis, and molecular lineage geographical analysis. Chemical identification research on Chinese medicine is a method that targets the analysis of characteristic chemical compounds in Chinese medicine materials.

### Processing method verification

Most of the Chinese medicine used in the CCF have been processed (*Paozhi*). *Paozhi* refers to the processing medicinal materials, which allow them to store easily, meet their clinical needs and improve their clinical efficacy. Most of the processing methods that were recorded in those 100 CCF are specifically stipulated in the ChP. However, more research is needed to study the processing methods that are not regulated or not consistent with the methods in the ChP. The main processing method in the CCF is to remove the unrelated parts of the medicinal materials. Simultaneously, slicing and the use of additional material processing (with auxiliary materials) are also widely used in the processing of CCF. The processed medicinal materials are called decoction pieces, whereas the unprocessed medicinal materials are called fresh drug. The existence of fresh drugs reflects the diversity of *paozhi* methods and the distinctive characteristics of prescription in CCF. For toxic Chinese medicine materials, the control of their toxic components is achieved through *Paozhi*, but more proofs are still needed for the safety use of toxic Chinese medicine materials [19].

*Paozhi* technologies are widely recorded in ancient medical books. However, some of them contain unclear information, confusing words, and non-accurate records, so some double-textual research or more research are needed to resolve these problems to standardize the specific details for processing [20]. Besides, there are only a few descriptions on the quantification of the *Paozhi* technologies in the ancient records. Even though there are some descriptions, most of them are subjective judgments, quantifying the *Paozhi* technologies is a big challenge in the research field. The same *Paozhi* technologies used may have different processing steps, so textual research on *Paozhi* should also compare the medicinal experience from the ancient books in different dynasties. Simultaneously, looking at the effects of each medicinal material in the prescription can help to trace back the original method and clear purpose of the *Paozhi*, which can provide more reliable evidence for the *Paozhi* method verification of CCF. Take Dachengqi Decoction as an example, the function of this decoction is to regulate the gastrointestinal tract. After Dahuang is processed with wine, its medicinal properties can be changed, and the heat of the triple energizers can be removed, which help to restore the health of the gastrointestinal tract. Therefore, in this prescription, the original *paozhi* method of Dahuang should be processed with wine.

### Dosage verification

Dosage is an important factor influencing the safety and effect of CCF. The dosage and ratio between each medicinal material determine its clinical efficacy. As the measurement unit in ancient times is different from modern times, the standard dosage of each CCF cannot be determined directly. Therefore, it is necessary to verify the dosage to achieve the standardization of the current dosage, which is important to ensure the safety and stability of the current dosage for the development of CCF.

Dosage is a comprehensive reflection of the treatment concepts from the ancient clinical doctors, which passes on their unique therapeutic thoughts, medication habits and the historical background of the prescriptions. The main factors that determine the dosage include: (1) the type of disease determines the primary medicine and dosage used in the prescriptions; (2) the age and physical conditions of the patients determine the overall dosage; (3) the prescription dosage form also has special requirements for the dosage; (4) the historical factors and medication habits in ancient times have a direct impact on the dosage.

The CCF are derived from the ancient records in the medical books of the past dynasties. Most of them are intuitive descriptions of the subjects, dosages, preparation methods, and there is lack of accurate data. The verification of the dosage is the verification of the quantity of medicinal materials. Due to the shortcomings of TCM inheritance, the dosage of medicinal materials is often considered confidentially, so there are many difficulties in verifying the dosages of some CCFs. In addition, CCF have a relatively large time span, the dosage of their original prescriptions has changed due to some errors in spreading and modification by clinical physicians, and the measurement units used in different dynasties are different [21].

At present, the key information tables published by the National Administration of Traditional Chinese medicine in China (NATCM) provide the dosage information for 7 CCFs only. There are still no unified standard dosages for the other CCFs [22]. The current reference for dosage is mainly from the ancient measurement units, clinical prescriptions and general textbooks from colleges and universities. The research on dosage is mainly focused on three aspects: ancient records, physical objects and clinical experience. After verifying the usage and dosage of CCFs recorded in the ancient books and their representative monographs on measurements in different eras, the actual dosage in the original prescriptions is finally determined. Due to political, economic and cultural differences, geographical and climate changes in ancient and modern times, and differences in the human body conditions, the dosages used in the original formula can be adjusted according to the clinical needs. The material object, especially the object for measurement units, in the specific dynasty is an important clue to determine the dosage of CCFs from the ancient books. The measurement units stipulate the measurement standard and conversion of length, weight and capacity. By combining the corresponding conversions of measurements in different eras, CCF will be in line with their original appearance. However, due to many factors, such as changes in time, differences in measurement, medicine and body composition, it is difficult to achieve a unified dosage. The practical experiences of clinical physicians have great reference values for verifying the dosages of CCF. These experience can also be used for some CCFs that cannot be verified by ancient books, literatures, measurements, or some prescriptions that are difficult to implement after being verified by other methods.

### Preparation process and quality evaluation methods for MR

#### Preparation process of MR

The preparation of MR should be based on the key information of CCF released by the Chinese government and the contents of ancient books. The released key information of CCF is based on the textural research, clinical experience of CCF and feasibility of industrialized production. If there are no details on the processing and preparation methods, it is necessary to refer to the *Management Regulations of Traditional Chinese Medicine Decoction Room in Medical Institutions* (医疗机构中药煎药室管理规范). Meanwhile, it is necessary to seek opinions from the clinical physicians and consider the feasibility of industrialized production. It is also important to ensure that the processing method is suitable for large-scale industrial production. The CCFMR samples are generally concentrated extracts and dried products, but no auxiliary materials should be added. Low-temperature concentration, freeze-drying, suitable storage containers, storage conditions and other suitable methods should be selected to ensure the stable quality of the CCFMR samples during the research period.

#### MR quality evaluation method

**Fig. 3:**
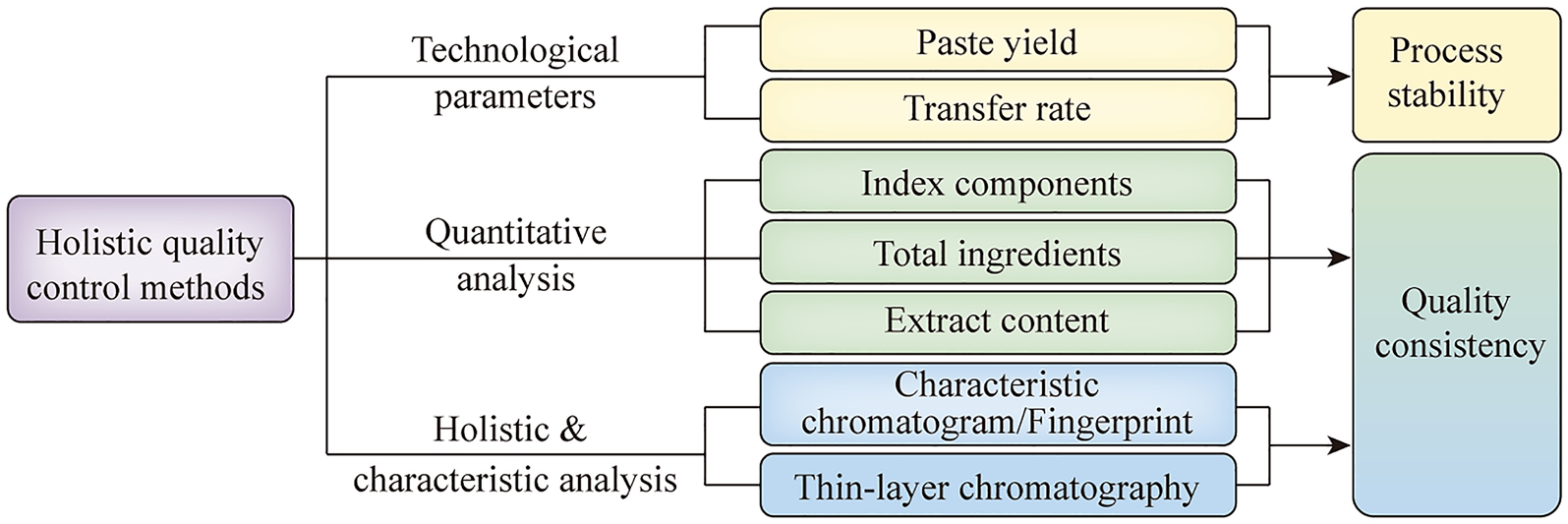
Holistic quality control methods for Chinese Classical Formula Material Reference (CCFPP). The quality of MR can be characterized in technical and product aspects, including the paste yield changes, transfer rate, multi-component measurement, specific and holistic identification

The quality of MR can be characterized in technical and product aspects, including the paste yield change, transfer rate, specific identification, multi-component measurement and holistic quality evaluation. Different methods, such as qualitative identification, quantitative determination of characteristic components and determination of total components, can be used to characterize each medicine in a CCF (Fig. 3). Among them, thin-layer chromatography and characteristic chromatogram have the characteristics of integrity and specificity, which are important methods for the identification of each medicine in a CCF.

Commonly used high performance liquid chromatography (HPLC) technology can provide a more comprehensive composition profile based on the MR. By optimizing chromatographic conditions, using stationary phases with different separation principles, and optimizing sample preparation methods, the quality analysis of each medicine in the CCF can be achieved. However, due to the inherent defects of some special medicinal materials, such as medicinal materials containing many volatile and fat-soluble ingredients, or medicinal materials with polysaccharides or amino acids as the main active substances [23], or medicinal materials with no specific standards in the existing pharmacopoeia, a single HPLC method cannot fully reflect the properties of all the compounds in the prescriptions, so it is necessary to use multiple detection methods for quality evaluation under multiple chromatographic optimization conditions [24].

The key quality attributes of the medicinal materials should be determined by correlation analysis of the medicinal materials, decoction pieces, intermediates, and their preparations. The pharmacological effect of components and the efficacy of the CCF are important factors for the selection of quality indicators for MR, which reflects the correlation between the quality of the preparations and their clinical efficacy [23].

Three rules were recommended in selecting crucial quality attributes or quality indicators for the quality evaluation of CCF effectively [12]: (1) Identification of all the flavors (each individual herb in a formula) to ensure that indicators can reflect the characteristic ingredients of each flavor in the CCF. (2) Measurement of the indicators in the key flavor of CCF to ensure the accurate ratio of the valuable medicine, namely the sovereign and minister flavors of a formula (君臣). (3) Presentation of adjuvant flavors (佐使) to ensure that the uniform quality of preparations is stable [14]. For sovereign and minister flavor and valuable flavor in the CCFPP, it is necessary to achieve quantitative control. However, precious medicine, such as ginseng, is only used in a small amount in the preparations, and the content of key components is too low for accurate quantification, but it can be qualitatively identified with its characteristic peaks.

## Development of CCFPP

With their background and clinical efficacy, CCF are the most important part of TCM due to its clinical efficacy, and it has drawn significant attention to many scientists. In April 2018, to implement the "TCM Law of the People's Republic of China" and promote the steady development of TCMPP derived from the ancient CCF, the State Administration of TCM in conjunction with the State Drug Administration promulgated the "Ancient Classical Formulas Directory" (ACFD) [5]. This directory covers hundreds of prescriptions since the Han Dynasty, and recruits a variety of ancient medical books, including "Treatise on Febrile Diseases" (伤寒论) and "Synopsis of the Golden Chamber" (金匮要略). The preparation forms include decoction (汤剂), boiled powder (煮散), powder preparation (散剂) and paste preparation (膏剂). Meanwhile, the selected prescriptions include more than ten traditional effects, such as clearing heat (清热), release the exterior (解表), purgation (泻下), harmonizing and releasing effects (和解). These selections in the directory have the characteristics of complete varieties, wide sources, and a variety of dosage forms. Therefore, in terms of R&D for CCFPP, more opportunities can be brought to the industrial companies, but it also requires in-depth thinking and careful selection.

Prof. Shilin Chen and colleagues concluded that a systematic investigation should be considered for the selection of CCF from three perspectives: drug evaluation, industrialization and clinical positioning [14]. They also proposed R&D ideas for CCFPP. Therefore, this provides a reference for the actual R&D and safety evaluation of CCFPP.

### Developmental strategy

#### Evaluation of druggability

Although the safety and effectiveness of CCF have been tested in clinical practice to a certain extent during the long-term application process, the registration policy clearly states that only the preparations with rigorous pharmaceutical researches and pre-clinical safety evaluation studies can be approved by the simplified approval method for registration and can be exempted from clinical trials. This registration policy is for making CCF into high-quality, safe and controllable products for the improvement of the quality of TCM. Therefore, evaluating the druggability of CCFPP should be focused on its pharmaceutical and pre-clinical safety research.

According to the requirements of issued policies (Table 1), the development of CCFPP has strict requirements for pharmaceutical research, which includes the R&D of CCFMR and the R&D of CCFPP. Firstly, the CCFMR must be developed by the prescriptions and preparation methods published in the ACFD. Secondly, the pharmaceutical research of CCF prescriptions must be conducted according to the CCFMR to ensure that the critical quality attributes of CCFMR and preparations are consistent.

**Table 1:** Relevant policy documents for Chinese Classical Formulas pharmaceutical preparations

No	Time	Documents	Main contents
1	2008.01	Supplementary Provisions by the Administration of Registration of Chinese Medicines	Three principles, exempt from clinical trials, no certificate for new drugs
2	2015.08	Opinions on Reforming the Review and Approval System for Drugs and Medical Devices	Simplified the approval process
3	2016.02	Outline of the Strategic Plan for the Development of Traditional Chinese Medicine	Encourage the development of classical Chinese medicine prescriptions
4	2016.12	Law of the People's Republic of China on Traditional Chinese Medicine	Article 30: Comply with national regulations and provide non-clinical safety research data
5	2017.03	The Selection Scope and Selection Principles of the Formulation of the Ancient Chinese Classical Formulas (CCF) Catalog	4 Selection principles
6	2017.10	Opinions on Reforming the Review and Approval System and Encouraging the Innovation of Drugs and Medical Devices	Article 13: Review and approve in accordance with simplified standards
7	2017.10	Simplified Registration and Approval Management Regulations for Chinese Classical Formulas pharmaceutical preparations (CCFPP) (draft for comments**)**	Simplified the approval process for CCFPP
8	2017.10	Application data requirements for standard decoction of Chinese Classical Formulas pharmaceutical preparations (draft for solicitation of comments)	Standard decoction related information should be provided
9	2017.10	Requirements for the application materials of Chinese Classical Formulas pharmaceutical preparations (draft for soliciting comments)	Exempt drug effect tests and clinical trials
10	2017.10	Technical Guiding Principles for the Evaluation of Chinese Medicine Resources	Estimated consumption, potential risks and sustainable use measures
11	2018.04	The State Administration of Traditional Chinese Medicine, in conjunction with the State Drug Administration, announced the "Catalogue of Ancient Classic Prescriptions (First Batch)"	Release the first batch of CCF (100 CCFs)
12	2018.05	Simplified Registration and Approval Management Regulations for Ancient Chinese Classical Formulas pharmaceutical preparations	Material benchmark, key quality attributes
13	2019.03	Application Requirements of Ancient Chinese Classical Formulas pharmaceutical preparations and Substance Standards	File a data declaration request
14	2020.04	Special Regulations on Registration and Management of Chinese Medicines (Draft for Solicitation of Comments)	Accelerate the promotion of Chinese Medicine Innovation
15	2020.09	Classification of Chinese Medicine Registration and Filing Requirements	Ceased review and release the unified standard of "Classic Named Substance Standards"
16	2020.10	"Key Information Principles of Ancient Classical Prescriptions" (7 Prescriptions)"	Rendered related requirements of key Information
17	2020.11	Technical Guidelines for Research on Homogenization of Traditional Chinese Medicine	Ensure uniformity and stability of product
18	2021.04	Technical Guidelines for Pharmaceutical Research on Chinese Classical Formulas pharmaceutical preparations administered in accordance with the ancient famous prescriptions (Draft for Solicitation of Comments)	Technical Guidelines for Pharmaceutical Research on CCFPP

Although certain highly toxic medicinal materials that have been proved to be toxic by modern toxicology are restricted when selecting in the ACFD, some prescriptions still contain toxic medicinal materials, such as Aconite, Pinellia and Asarum. Meanwhile, as the requirements for clinical trials are exempted, the requirements for non-clinical safety research are even higher. The registration regulations clearly state that experiments must be conducted in a laboratory with GLP qualification, and they should strictly follow GLP specifications. Therefore, enterprises should be aware of the importance of non-clinical safety research during R&D processes.

The ACFD provided the ancient dosages, which cannot be clearly converted to the modern dosages and may not consider the safety and druggability of the prescriptions. There are two main disputes on the dosage conversion of "Treatise on Febrile Diseases", such as "1 Liang (两) = 13.8 g", and "1 Liang = 3 g". The difference in dosages is nearly five times. If one CCF is developed into an MR according to different dosages, it will lead to a phenomenon that the content of MR of the same CCF used by each enterprise is entirely different. Thus, this will further increase the difficulty in evaluating CCF and making it difficult to unify the standard of CCFMR [14]. Therefore, it is recommended to select an appropriate dose during R&D and conduct related safety tests under GLP specifications.

#### Industrialization considerations

The basic principle of the industrial production of CCFPP is to produce modern preparations with the same quality as traditional preparations. Its focus is to establish a manufacturing process that is reasonable, low cost, stable and controllable, and to produce products with quality that is biologically and chemically consistent with the CCFMR. The industrialization considerations of CCFPP mainly involve the production process, and control of quality influencing factors and production cost. In actual industrial production, many problems from the extraction process, solid–liquid separation, concentration, drying and formulation processes may be encountered. Among them, the main factors affecting the quality of CCFPPs are the quality of raw materials, extraction process parameters, and industrialization of powder (散剂) and paste (膏剂) preparations. The prerequisite for the stable quality of CCFPP is that the quality of the decoction pieces used in the prescriptions should be uniform and stable. Mixed batch feeding of decoction pieces may be a feasible way to ensure the uniform and stable quality of CCFPPs. The production costs of Chinese medicine are mainly composed of raw medicinal material and auxiliary material costs, packaging material cost, direct labor, fuel engine power, and manufacturing cost. Among them, direct material and labor costs account for more than 50% of the production costs. Achieving effective control on raw material and labor costs is the key target to improve cost control and management.

#### Clinical positioning

CCF should not be developed into the type of drug that could cure all types of diseases, because this is not conducive to the precise clinical use of CCF and this cannot fully reflect their advantages. A broad range of clinical positioning can lead to unreasonable clinical use, which is one of the main reasons that can affect the efficacy of Chinese medicine. It does not only affect the efficacy of Chinese medicine, but also leads to the repeated occurrence of adverse reactions and even toxic effects from Chinese medicine [25–27]. For example, "Xiao Chai Hu Tang incident" in Japan is an example of misuse due to unclear clinical positioning [28]. If the clinical positioning is accurate, the toxic and side effects of Chinese medicine can be minimized, and their therapeutic effects can also be maximized.

The clinical positioning of the CCF refers to the re-evaluated clinical efficacy of listed Chinese patent medicine. Firstly, the clinical use of CCF should be sorted out systematically in the records from clinical physicians in the past dynasties. Meanwhile, modern research and application of CCF should be collected, sorted and summarized. The systematic evaluation method of the evidence-based medicine is then used to analyze and evaluate CCF one by one to provide a clinical basis for the layout of CCF. Clinical indicators are primary selected for each species. Modern pharmacological and toxicological research results of the prescriptions are then analyzed and summarized to provide references for current research. The scope of clinical positioning of the prescriptions should be narrowed. Finally, the clinical characteristics of the prescriptions should be summarized, the main therapeutic effects should be clarified. They should also consider the secondary effects of the prescriptions, pay attention to their side effects, and confirm its clinical positioning after multidisciplinary approaches, especially on the demonstration from clinical experts [14].

### Processes for developing CCFPP

**Fig. 4:**
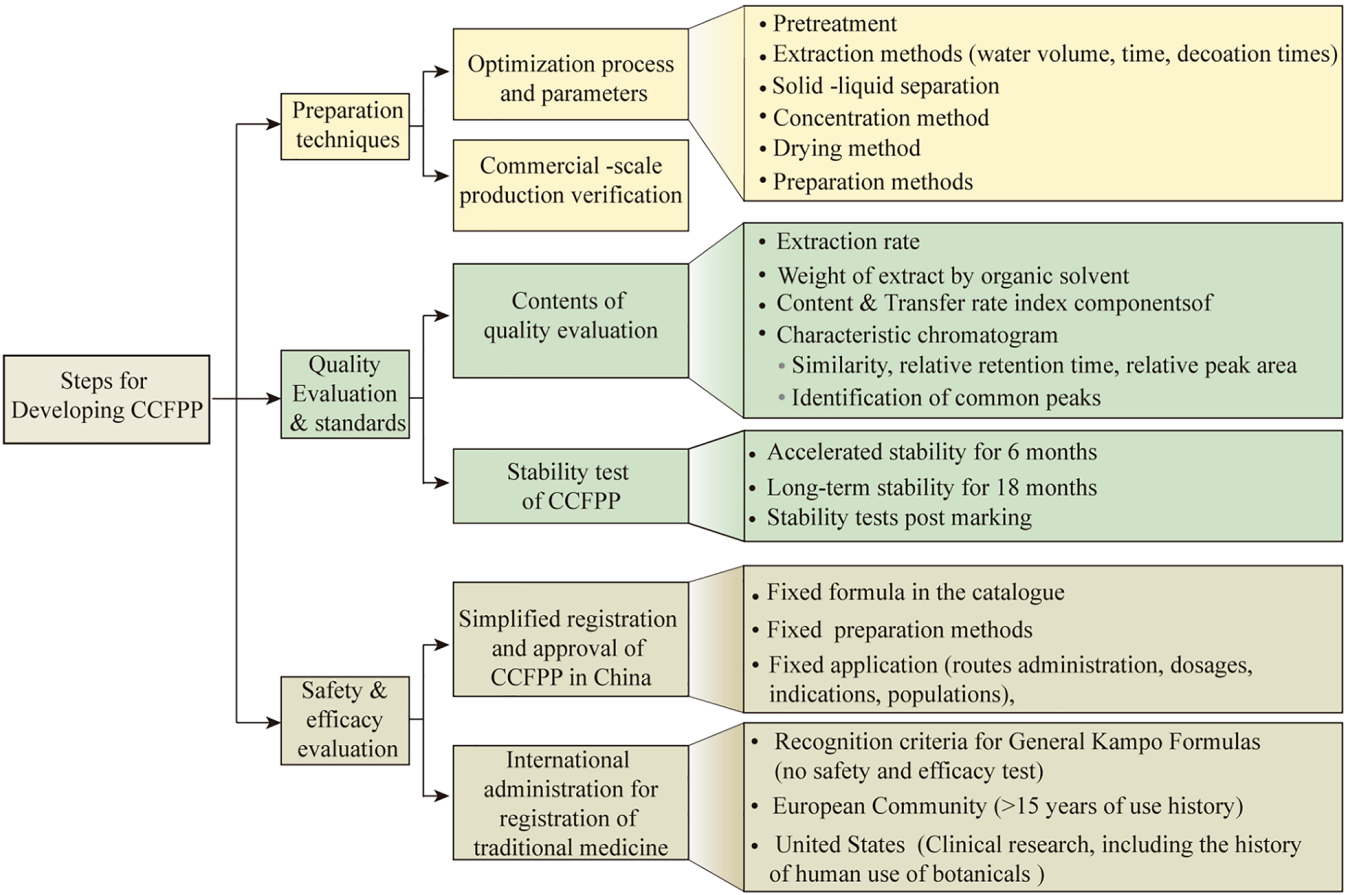
Critical steps for developing Chinese Classical Formula pharmaceutical preparations (CCFPP). There are three main steps in the development of CCFPP, optimization of the preparation methods, development of the quality analysis methods and quality standards, and safety evaluation

There are three main steps in the development of CCFPP, preparation method optimization, quality analysis method development and quality standard, and safety evaluation (Fig. 4). The content of each step is elaborated below.

#### Preparation techniques

The research of CCFPP should aim at producing CCFPP with same quality as the CCFMR. The research contents include studying and determining the technical processes and parameters (ranges) of the pre-treatment, extraction, solid–liquid separation, concentration, drying and preparation molding of CCFPP. Furthermore, it should complete commercial-scale production process verification and draw up detailed operating procedures. Due to the limitation of historical technologies, the traditional preparation methods of CCF are relatively rough and individualized. The development of new drugs is based on the large-scale industrial production. Many parameters in the manufacturing process can be gradually optimized in the pilot study to achieve the goal that the key quality attributes of the CCFPP are consistent with the those determined by the CCFMR, while realizing the rational use of Chinese medicine resources. In the pilot research and large-scale industrial production, water should be kept as the extraction solvent for decoction. Other preparation processes can be changed according to the requirements of large-scale production equipment, such as the amount of water, the number of extractions, the extraction time, the filtration method, the concentration method, the drying method, and the preparation method.

Quality is another important factor for optimizing the preparation process to ensure process stability and reduce the fluctuation range of preparations. Clarifying the upper and lower limits of the control range, including paste yield, transfer rate and other indicators of process, is important to ensure the stability of the preparation production process. It is also important to ensure the uniformity and stability of the preparation quality. The relevant administrative departments in China stated that the acceptable fluctuation range of the main testing indicators for the preparation quantity is 70–130% in average. For indicators outside this range, it is required to analyze the reason by correlation analysis between medicinal materials, decoction pieces, preparation intermediates and CCFPP.

The composition of Chinese medicine is complex, and its therapeutic effects are the joint effects of multiple components. When the composition ratios of different components are the same or similar, their therapeutic effects will be similar. Therefore, when exploring the process parameters for CCFPP, the composition ratios between different components must be compared. Importantly, the composition ratios should be consistent with the CCFMR.

#### Quality evaluation and stability test

The holistic quality standard of the preparation is important to measure the quality of the product. The methods of quality control should strengthen with specific identification and multi-component quality control. It should at least include the extraction rate, quantitative determination of the characteristic components and their transfer rate, and fingerprint/characteristic map. The fingerprint/characteristic map should clarify the requirements of similarity, relative retention time and relative peak area. The main components should be identified as much as possible in the fingerprint/characteristic map. At the same time, factors related to safety should be paid attention.

**Fig. 5:**
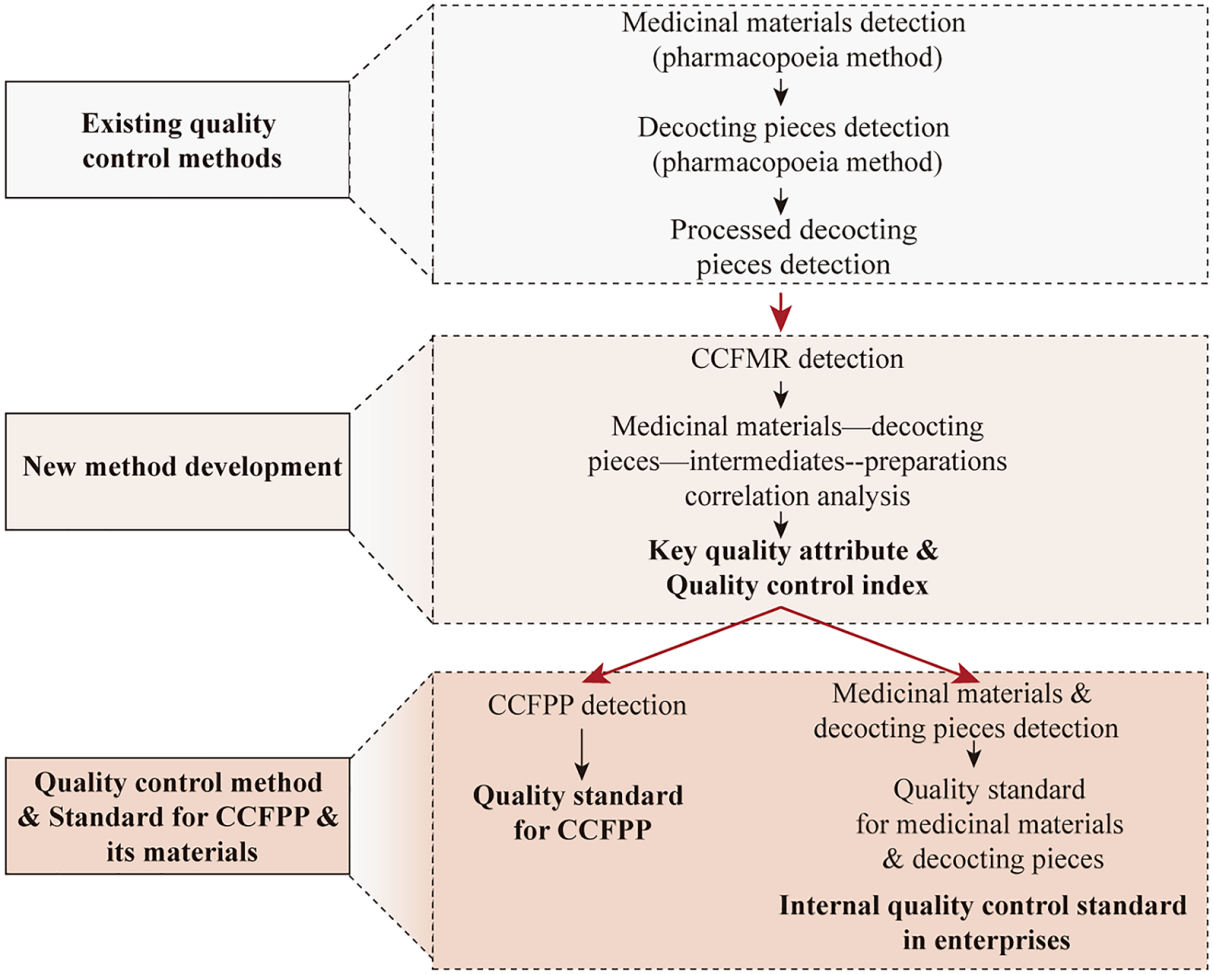
The development process of holistic quality control methods for Chinese Classical Formula pharmaceutical preparations (CCFPP) based on the whole manufacture process. The holistic quality control methods include the quality control from medicinal materials—decoction pieces Chinese Classical Formula pharmaceutical material reference (CCFMR) — Chinese Classical Formula pharmaceutical preparations (CCFPP) with key quality control index

A scholar Prof. Dai proposed that the development process of quality control methods and standards for CCFPP can be divided into three steps (Fig. 5) [24]. Firstly, the medicinal materials and decoction pieces are tested according to the existing quality evaluation methods and standards in the ChP to select the qualified raw materials. Secondly, with the CCFMR as the object, a holistic quality control method should be developed, which can detect all the medicinal materials in the formula. The key quality attributes and key quality indicators can also be selected by correlation analysis of the medicinal materials, decoction pieces, intermediates and CCFPP. With the key quality attributes, holistic quality control methods can be established for CCFPP, and its quality standard can be established according to the quality data of different batches of CCFPPs. The medicinal materials from which the index components are derived from can be confirmed by correlation analysis between CCFMR and decoction pieces. If the determined quality indicators/index is different from the index components in the existing quality evaluation methods of the medicinal materials in the ChP, a new quality control method with the new index component for medicinal materials and decoction pieces should be developed by the third step. Finally, with the determined quality indicators, optimized quality control methods for raw materials and decoction pieces should be developed. With the new analysis methods, decoction pieces and medicinal material are analyzed to determine the required quality standards of decoction pieces to meet the standards of CCFPP [23]. These new quality control methods and quality standards of raw materials and decoction pieces can be used as internal quality control methods and standards in enterprises to ensure the quality of CCFPP.

The validity period of CCFPP should be determined based on the results of long-term test. In general, the data of 6-month accelerated stability test and 18 months long-term stability test should be provided. After the drug is put on the market, the stability test should be continued.

#### Non-clinical safety evaluation

After long-term clinical practice tests and accumulated experiences, prescriptions with poor efficacy and safety have been gradually eliminated, while CCF with safety and definite efficacy have been used until today. To enable CCF to meet the needs of patients, the NMPA in China have issued relevant regulations, clarifying that “for CCF preparations that meet the requirements, they can provide pharmacy and non-clinical safety research data without reporting pharmacological research and clinical trial data". This is aimed to simplify the registration and approval procedures to promote the development of Chinese medicines. However, there are concerns about this as simplified registration may reduce the requirements for drug safety and lead to medication risks.

##### Safety evaluation for simplified registration and approval of CCF

CCF are derived from ancient medical records and have a long-term basis from clinical application. The safety and effectiveness have been confirmed by clinical practice, so it is reasonable to implement simplified registration and approval for them. Misuse of medicinal materials, improper processing, unreasonable long-term medication, improper compatibility and inconsistent use of medicine are the main reasons for the occurrence of adverse reactions in recent years [21]. To avoid the potential safety problems caused by the simplified registration and approval, the NMPA in China has established regulations on the selection of prescriptions and has also set requirements for simplified registration of CCF, including formulations, preparation methods, routes of administration, dosages of drugs, therapeutic effects and indications, applicable populations, and qualifications of manufacturers in many aspects. Besides, each CCF preparation needs to conduct non-clinical safety studies that follow GLP standards to systematically, objectively and comprehensively evaluate the safety of CCFPP to ensure the safety of clinical medications. In this case, only the prescriptions listed in the ACFD are eligible for simplified registration and approval. The prescriptions selected in this directory should meet the requirements of "widely used, definite therapeutic effects, obvious characteristics and advantages, sufficient records and evidence in medical records from ancient times, sufficient clinical and experimental research reports in modern literatures, further clinical studies of Chinese medicine and widely recognized by authoritative experts, described in various Chinese medicine textbooks" and other requirements.

##### The context of safety evaluation for CCFPP

The long-term clinical application and reasonable formulation of the CCF could reduce the toxicity of Chinese medicine to achieve relatively mild effects. However, due to the use of many new technologies in the modern Chinese medicinal preparations, there are significant changes in the routes of administration, dosage forms and material bases. Some changes are unknown, especially when the contents of key ingredients are significantly increased, their pharmacological effects may be significantly enhanced, and the adverse reactions may also be increased. Therefore, CCFPPs should be tested for safety evaluation, including acute and long-term toxicity studies.

The pre-clinical safety evaluation of the CCFPPs can be selectively conducted according to the specific conditions of the drug itself. For example, the safety test can be conducted as needed, and the toxicity of a single dose can be tested in a single type of animal. For toxicity test, repeated doses test can be firstly conducted with repeated doses in one type of animal (rodent). When toxicity is found, further studies are needed to test the toxicity with repeated doses in other kinds of animals (non-rodent). Special toxicity tests, such as genetic toxicity test, reproductive toxicity test, carcinogenic test and dependence test, are all needed when evidences suggest that they should be conducted (preliminary experimental results and literature reports). This requires a systematic summary and evaluation of the current safety information of the prescriptions before conducting the research. Therefore, making an experimental design and a research plan are necessary and should be based on the characteristics of the prescriptions.

##### International administration for the registration of Traditional herbal medicine

"World Health Organization (WHO) Traditional Medicine Strategy 2014–2023" by the WHO pointed out that Traditional and alternative medicine can be found in almost every country around the world, and its demand is growing. Herbal medicine is essential for the implementation of Traditional medicine. As its safety and effectiveness have been confirmed to a certain extent in ancient times, many countries and regions have given specific simplified requirements for the application of registration of Traditional herbal medicines according to their respective circumstances.

The recognition criteria for General Kampo Formula is the basis for the research and production of Kampo medicine in Japan. Under this circumstance, within the range mentioned in the regulation, when only water is used as the extraction solvent, any companies can independently determine the compatibility of medicinal materials, select dosage forms, formulate process and quality standards, and directly produce the products without pharmacological and clinical researches [10]. For Kampo medicine that was not included in the “Recognition criteria for General Kampo Formula” but was approved for production from 1968 to 2015, other companies only need to conduct the process and quality standards studies when applying for production without the needs of providing pharmacological and clinical research data if there are no added solvents other than water in the formulas.

According to the characteristics and uses of Traditional herbal medicine in European Union countries, the European Parliament stipulates that registered enterprises within the European Community can be exempted from conducting clinical and pre-clinical experiments when applying for the registration of Traditional herbal medicine if the products can meet the listed requirements, including oral, topical or inhaled Traditional herbal preparations that do not require clinical physicians to diagnose, prescribe and supervise with certain specifications and dosages. The medicine needs to have at least 30 years of medication history before the registration, including at least 15 years of use history in the European Community, sufficient data showing the traditional and safe use of the product under certain conditions, and confirmed pharmacological efficacy [19].

In the United States, the United States Food and Drug Administration (FDA) allows the application of new medicine under the over-the-counter drug (OTC) monograph system or new drug application (NDA) if their safety and effectiveness are approved as some botanical products and they have been used for a long time in the United States. However, clinical research data are needed for the application for botanical drug marketing under the OTC monograph system and NDA application system. Among them, the safety information provided by the human use history of botanicals should also be considered. Considering the characteristics of botanical medicine, the United States FDA reduces the technical requirements for the first clinical trials, so for most botanicals at this stage, the characteristics of the botanicals (chemical composition, manufacturing and quality control) are not required for guarantee. Simultaneously, for the plant products that are legally listed on the market as supplementary food, pre-clinical pharmacological and toxicological research are also not required when applying for the first clinical trials if there are no safety issues and the dosage is roughly the same [20].

##### Pharmaceutical research and safety

Following the requirements of Chinese 6.1 categories new medicine from *Drug Regulation Management Measures* and other related policies, toxicological research is needed to provide 7 types of experimental research data: (1) Summary of toxicological research data. (2) Acute toxicity test data and literature reviews. (3) Long-term toxicity test data and literature reviews. It is also necessary to consider whether the following research data are needed according to the specific circumstances of the research project: (4) Safety test data and literature reviews on allergy (local, systemic and photosensitive toxicities), hemolytic and local irritation and dependency (vascular, skin, mucous membrane and muscle) that are related to local and systemic administration. (5) Genetic toxicity test data and literature reviews. (6) Reproductive toxicity test data and literature reviews. (7) Carcinogenicity test data and literature reviews. Considering the characteristics of CCF and strict screening procedures, only 1–3 types of experimental research data are required under normal circumstances. If necessary, 1–3 more types of individual experimental research data or all other experimental research data should be supplemented.

## Conclusions

As a vital part of the clinical prescriptions, CCF are the primary support for all the prescription science and even for the theoretical system of TCM. The long-term practice has provided evidences to show their clinical efficacy and low safety risks. Combining ancient and modern applications and standardizing their preparation methods are key considerations for the development of CCF. Expert consensus might also be a good approach to solve the major issues of the modernization of CCF, including the origin of the medicinal materials, processing methods, dosages, and preparation processes. The modernization of CCF by the pharmaceutical industry has become mature, the R&D of CCF is expected to promote the development and improvement of the theoretical system of prescriptions and benefit more to the patients in a broader range.

## Data Availability

Not applicable.

## Abbreviations

ACFD: Ancient Classical Formulas Directory
FDA: Food and Drug Administration
CCF: Chinese Classical Formulas
COVID-19: Coronavirus disease 2019
GLP: Good Laboratory Practice
HPLC: High performance liquid chromatography
MR: Material Reference
PP: Pharmaceutical preparation
NDAs: New drug applications
OTC: Over-the-counter drug
R&D: Research and development
TCM: Traditional Chinese medicine
WHO: World Health Organization
NMPA: National Medical Products Administration in China
NATCM: National Administration of Traditional Chinese medicine
